# Transfer learning-driven prediction of oil and gaspipeline corrosion rates in small sample scenarios

**DOI:** 10.1371/journal.pone.0333914

**Published:** 2025-10-16

**Authors:** Pujun Long, Guopan Yang, Haiyang Liu, Weiwei Deng, Mi Liang

**Affiliations:** 1 School of Mathematical and Physical Sciences, Chongqing University of Science and Technology, Chongqing, China; 2 School of Artificial Intelligence, Chongqing University of Science and Technology, Chongqing, China; KFUPM: King Fahd University of Petroleum & Minerals, SAUDI ARABIA

## Abstract

To ensure the safe operation of oil and gas pipeline systems in complex environments, accurately predicting the corrosion rate of natural gas well pipes is of paramount importance. Given the widespread challenge of pipe corrosion in the oil and gas industry, we propose a transfer learning model based on a CNN-LSTM-Transformer architecture with a staged fine-tuning strategy for corrosion rate prediction under small-sample conditions. The model integrates Convolutional Neural Networks (CNN), Long Short-Term Memory networks (LSTM), and Transformer modules. CNN is employed to extract local features from corrosion data. LSTM captures the temporal dependencies within the data, and the Transformer module applies multi-head attention to recalibrate features, effectively addressing long-range dependencies. To enhance the model’s adaptability, the CNN-LSTM-Transformer model is initially trained on a source domain and then progressively fine-tuned on a target domain to facilitate knowledge transfer. Experimental results demonstrate that, after staged fine-tuning, the CNN-LSTM-Transformer model achieves an MAE of 0.021, RMSE of 0.031, and an R² of 0.909, outperforming other transfer learning approaches by a substantial margin.

## Introduction

Oil and gas pipelines that have been in use for extended periods are prone to corrosion, particularly long-distance pipelines, where the issue becomes even more severe. This not only affects the structural integrity of the pipeline but also results in leakage or explosions, posing significant risks to human life, property, and the ecological environment [1]. Therefore, accurate prediction of pipeline corrosion rates and effective control of corrosion are necessary.

Currently, there are three main methods for corrosion prediction in oil and gas pipelines: empirical models, statistical models, and machine learning models. Empirical models are corrosion prediction methods built based on practical experience and historical data [2]. Although these models are effective under specific conditions, their applicability and accuracy may be limited under different conditions due to an excessive reliance on empirical data. Statistical models use mathematical and statistical methods to analyze data and identify trends and patterns in the corrosion process [3]. These models typically assume the existence of linear relationships or specific statistical distributions in the data and rely on predefined relationships between variables. However, the pipeline corrosion process often involves complex nonlinear and multivariable interactions, which may not be adequately captured by relying solely on statistical models.

To address the limitations of empirical models and statistical models, researchers have proposed machine learning models [4,5]. Machine learning models can learn complex patterns and relationships within data, automatically identifying nonlinear and high-dimensional features. This enables machine learning models to exhibit greater adaptability and predictive accuracy when dealing with varying conditions and diverse datasets. For example,Soomro et al. [6] evaluated the application methodology of Bayesian network models and relevant influencing factors in pipeline corrosion rate prediction. Ren et al. [7] developed a prediction model for internal corrosion rates in natural gas pipelines by applying Back Propagation(BP) neural network to long-distance pipeline corrosion rate prediction. El-Abbasy et al. [8] using historical data from existing pipelines in Qatar, developed a condition prediction model for non-piggable oil and gas pipelines through regression analysis, Artificial Neural Networks (ANN), and decision tree models. This model enabled the evaluation of pipeline operational conditions. Kim et al. [9] developed a duplex corrosion depth prediction model based on improved comprehensive fuzzy evaluation, which can characterize multiple corrosion characteristics under varying environmental conditions. Peng et al. [10] improved Genetic Algorithm and Particle Swarm Optimization to propose an optimized Support Vector Regression for solving parameter selection problems. By combining this with the unequal interval grey prediction method, they developed a gas pipeline corrosion prediction model with higher accuracy than traditional models. Shaik et al. [11] employed ANN models to predict the service life conditions and metal loss failure classifications for five pipeline segments in Sudan’s oil and gas industry.

Although the aforementioned methods have made significant progress in both theory and practice, their effectiveness and accuracy largely depend on sufficient high-quality data. However, these data are difficult to obtain, as real-time monitoring of corrosion states requires high-precision, complex, and expensive equipment and technologies. Moreover, oil and gas pipelines typically operate in environments with fluctuating temperature, pressure, and chemical composition, making data collection and standardization challenging. Furthermore, many oil and gas pipelines lack systematic corrosion monitoring during operation, resulting in insufficient historical data. Data sharing among different companies is also frequently restricted by trade secrets and privacy protection, further reducing the amount of available data.

In recent years, Transfer Learning (TL) has been widely applied in Computer Vision, Text Classification, Activity Recognition, and Multilingual Speech Technologies [12–14]. By leveraging knowledge from a source domain to address challenges in a target domain, this approach has demonstrated remarkable efficacy, even in scenarios with limited or entirely absent labeled data. Beyond these domains, TL is increasingly recognized for its transformative potential in engineering applications. In particular, it has proven to be a powerful tool for addressing the pervasive issue of data scarcity in real-world engineering tasks [15].

Xiao et al. [16] proposed a TL based fault diagnosis framework that combines the modified Transfer Adaboost algorithm with Convolutional Neural Networks(CNN), addressing the issue of insufficient small sample data in mechanical fault diagnosis. Luo et al. [17] proposed a TL method based on the Compact Lookahead Model. This method combines feature mapping, feature learning, and domain adaptation. It aims to address the issue of insufficient samples in the quality analysis of small bolt fastening data. Zou et al. [18] proposed a TL method based on a CNN-LSTM fusion model to address the small sample problem in lithium-ion battery performance evaluation. Wang et al. [19] proposed a novel hybrid TL method aimed at addressing the challenges faced in high-voltage circuit breaker fault diagnosis under small sample conditions.Xiao et al. [20] established a TL based model combining CNN and BP Neural Networks to address the fatigue life prediction problem of corroded bimetallic steel bars under small sample conditions. Ji, H. et al. [21] developed a machine learning model based on the transfer learning paradigm in complex natural environment scenarios, which improves the predictive ability of corrosion rates of steel in concrete by transferring knowledge of steel corrosion under laboratory conditions.

Given the need for accurate corrosion rate prediction to ensure the safe operation of oil and gas pipeline systems and the challenge of small-sample modeling in the industry, this study aims to achieve high-precision prediction of corrosion rates in natural gas well pipes under small-sample conditions by constructing a CNN-LSTM-Transformer TL with a staged fine-tuning strategy, thereby enhancing the model’s cross-domain knowledge transfer capability.Considering the effectiveness of TL in solving small sample problems, this study designs a TL model incorporating CNN-LSTM-Transformer architecture. In this model, CNN is used to extract local spatial features from pipeline corrosion data, while Long Short-Term Memory (LSTM) captures dynamic characteristics of sequential data. The Transformer module further enhances the model’s capability to capture long-range dependencies and express global features through its Multi-head Attention (MHA). Through this integration, the model can effectively utilize source domain data for pre-training under the TL framework. By employing a Gradual fine-tuning strategy to adapt to target domain features, the model achieves high precision prediction of pipeline corrosion rates with limited sample data.

The paper commences with a methodological exposition of the proposed corrosion rate prediction framework, which synergistically integrates Convolutional Neural Networks, Long Short-Term Memory, and Transformer modules, enhanced by a progressive fine-tuning strategy. This is followed by a detailed delineation of the transfer learning mechanism, emphasizing cross-domain feature adaptation under limited data conditions. Empirical validation is then carried out through two sets of benchmark experiments designed to rigorously evaluate model performance and generalization capability. The study concludes with a synthesis of key contributions and insights into the practical implications of the proposed approach for predictive maintenance in pipeline infrastructure.

## Corrosion rate prediction based on CNN-LSTM-Transformer and Gradual fine-tuning strategy

### Global technical framework

**Fig 1:**
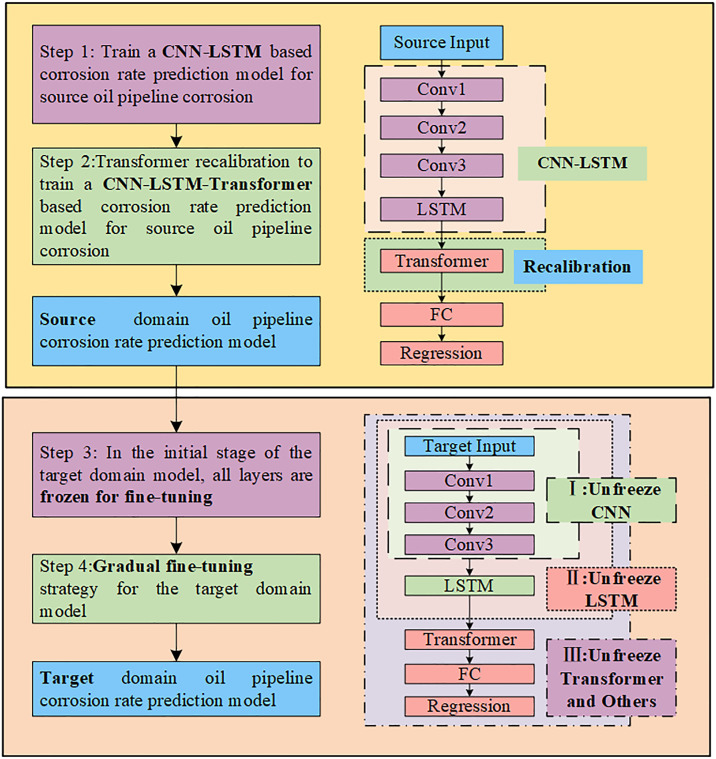
Architectural overview of the proposed method.

This paper proposes an integrated oil and gas pipelines corrosion rate prediction method incorporating CNN-LSTM-Transformer model and Gradual fine-tuning strategy. The overall framework is illustrated in Fig 1. The model consists of two main phases: In the first phase, a CNN-LSTM pipeline corrosion rate prediction model is constructed based on source domain pipeline corrosion data. Considering the limitations of conventional LSTM models in capturing long-range dependencies, we introduce the Transformer mechanism for feature recalibration upon this foundation. In the second phase, Gradual fine-tuning is performed on the pre-trained model using target domain pipeline corrosion data.

This process can be further divided into five steps:

Step 1, **Source Domain Model Construction:** Initially, a CNN-LSTM feature extraction model for pipeline corrosion rate prediction is constructed. In the experimental setup, corrosion datasets are designated as source domain dataset A and target domain dataset B. The pre-training model is trained using dataset A to provide initial weights for the target domain task.
Step 2, **Transformer Recalibration:** To address the limitations of conventional LSTM models in capturing long-range dependencies, a Transformer mechanism is introduced for feature recalibration. Extended features are processed through MHA to capture long-range dependencies. This process encompasses: computing MHA to obtain weighted representations for each input time step. Regularization is implemented through Dropout and Layer Normalization to prevent overfitting. The final pre-trained CNN-LSTM-Transformer model is constructed and trained using dataset A, with model performance evaluated through 5-fold cross-validation.
Step 3, **Target Domain Model Construction:** For target domain dataset B, a Gradual fine-tuning strategy is implemented on the CNN-LSTM-Transformer model. The adopted CNN-LSTM-Transformer architecture consists of three CNN layers, one LSTM layer, and a Transformer module. In the initial stage of model fine-tuning, parameters of all layers are frozen to maintain the stability of pre-trained weights.
Step 4, **Gradual Fine-tuning Strategy:** the process follows a Gradual sequence. Initially, the CNN component of the feature extraction model is unfrozen, allowing fine-grained adjustment of local features on target domain data. Subsequently, the LSTM layers are unfrozen to optimize their capability in modeling long-range dependencies for target domain data. Finally, the Transformer structure and subsequent Dense layers are unfrozen to further calibrate global feature representations and enhance ultimate prediction performance.
Step 5, **Target Domain Model Results:** Following the completion of Gradual fine-tuning, the refined model is employed to generate pipeline corrosion rate predictions on target domain data.

### Feature extraction

#### CNN-LSTM.

CNN demonstrates powerful feature extraction capabilities and superior performance [22]. Through local connectivity, weight sharing, and pooling operations, it effectively captures local features and spatial gradual structures in input data. Pipeline corrosion data contain complex sequential features and local patterns. The multi-layer structure of CNN progressively refines features through its gradual layers, enhancing the prediction capability of corrosion rate. Therefore, in designing the pre-training network, CNN layers are initially employed to extract influential features of pipeline corrosion rate from the source domain. The CNN component in this study comprises three convolutional layers, which are followed by max-pooling layers with a window size of 2.

The convolution kernel evaluates and extracts different features from various aspects of the data through convolution operations. For input data $\text{X}\in R^{m\times n\times \text{1}}$, and each sample $X_{i}(i=\text{1},\text{2},\ldots ,m)$ in the input matrix, the specific computation is shown in Equation (1).


$$\begin{array}{c} \begin{array}{c} X_{i}^{l}=\sigma \left(W_{i}^{l}{\;*\;X}_{i}^{l-\text{1}}+b_{i}^{l}\right) \end{array} \end{array}$$ (1)

where $\text{X}\in R^{m\times n\times \text{1}}$ represents: m is the number of samples, n denotes the number of features per sample, and 1 indicates the number of feature channels(this is 1-dimensional data).σ is the activation function, $W_{i}^{l}$ represents the weight matrix of the i−th convolution kernel in layer i, the symbol * denotes convolution operation, and $b_{i}^{l}$ is the bias term. The first convolutional layer employs 256 convolution kernels with a kernel size of 2. Each convolutional layer is followed by a max-pooling layer, where the pooling kernel scans the feature matrix output from the convolutional layer in a sliding window manner, thereby reducing the spatial dimensions of the feature map. In this study, a pooling window size of 2 is used, meaning that the maximum value is taken from every two adjacent elements, thus halving the length of the feature map. The specific calculation is shown in Equation (2).


$$\begin{array}{c} \begin{array}{c} \begin{array}{c} Y_{i}^{l+\text{1}}(j)=max\left\{X_{i}^{l}(k)\right\}\\ k\in D_{j} \end{array} \end{array} \end{array}$$ (2)

where ${\text{X}}_{\text{i}}^{\text{l}}(\text{k})$ represents the elements within the pooling kernel region in the i−th feature matrix of layer l, ${\text{Y}}_{\text{i}}^{\text{l}+\text{1}}(\text{j})$ denotes the elements in the i−th feature matrix of layer l+1 after the pooling operation, and ${\text{D}}_{\text{j}}$ represents the region covered by the pooling kernel on the convolution output.

Through pooling operations, the model preserves essential features while reducing computational costs and enhancing robustness to interference. The second and third convolutional layers employ 128 and 64 convolution kernels respectively, with the second layer using a kernel size of 2 and ReLU activation function. After three convolutional layers and two pooling layers, the CNN component extracts local spatial features. The convolutional output is transformed into the LSTM sequence input format as Y.

In the corrosion rate prediction task, sequential dependency of data is crucial. Therefore, following the CNN component, LSTM layers are introduced to capture sequential dependencies in the data [23]. The output Y is fed into LSTM layers, producing hidden states ${\text{h}}_{\text{t}}\in R^{\text{H}}$, and H=50.

LSTM comprises input gate ($i_{t}$), forget gate ($f_{t}$), output gate($o_{t}$) and cell state update ${\tilde{C}}_{t}$. Its calculation process can be shown by the following equation.

(1) Forget gate

$$\begin{array}{c} f_{t}=\sigma \left(W_{f}\left[h_{t-\text{1}},Y_{t}\right]+b_{f}\right) \end{array}$$ (3)

where $h_{t-\text{1}}$ denotes the hidden state at time step t−1, $Y_{t}$ denotes the feature vector of Y at time step t, the weights and biases of the unit state are $W_{f}$ and $b_{f}$.

(2) Input gate and candidate cell state

$$\begin{array}{c} i_{t}=\sigma \left(W_{i}\left[h_{t-\text{1}},Y_{t}\right]+b_{i}\right) \end{array}$$ (4)

$$\begin{array}{c} {\tilde{C}}_{t}=tanh\left(W_{c}\left[h_{t-\text{1}},Y_{t}\right]+b_{c}\right) \end{array}$$ (5)

where the weights and biases of the unit state are $W_{i}$,$W_{c}$ and $b_{i}$,$b_{c}$.

(3) Cell update state

$$\begin{array}{c} C_{t}=f_{t}C_{t-\text{1}}+i_{t}{\tilde{C}}_{t} \end{array}$$ (6)

where $C_{t-\text{1}}$ denotes the cell state at the previous time step t−1.

(4) Output gate and hidden state

$$\begin{array}{c} \begin{array}{c} o_{t}=\sigma \left(W_{o}\left[h_{t-\text{1}},Y_{t}\right]+b_{o}\right)\\ h_{t}=o_{t}\;\text{tanh}\left(C_{t}\right) \end{array} \end{array}$$ (7)

where the weights and biases of the unit state are $W_{o}$ and $b_{o}$, $h_{t}$ denotes the hidden state at the current time step.

**Fig 2:**
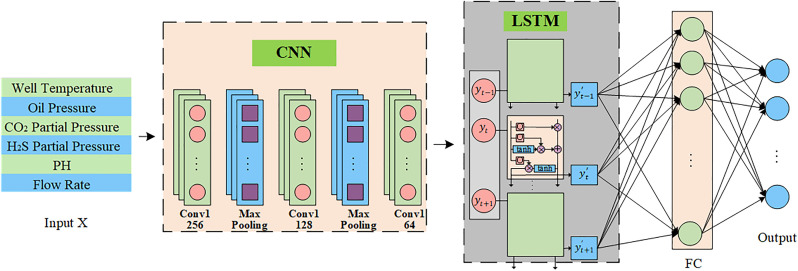
Structure of CNN-LSTM.

In this model, the LSTM layer has 50 hidden units with ReLU activation function and is followed by a Dropout layer (rate = 0.5) to prevent overfitting. The structure of CNN-LSTM is shown in Fig 2.

#### Transformer recalibration.

LSTM performs well in processing sequential data and can effectively capture both short-term and long-term dependencies. However, it has limitations in modeling long-distance dependencies due to information decay, vanishing gradients, and insufficient feature representation capability. Therefore, Transformer is introduced for feature recalibration to enhance model performance. The transformer’s parallel computing capability and self-attention mechanism effectively compensate for LSTM’s limitations. The combination of both achieves more accurate modeling of long-distance dependencies and significantly improves the overall processing capability for complex sequential data. The Transformer encoder is used for global recalibration of features provided by LSTM through MHA, Residual Connection and Layer Normalization (Add & Norm), and Feed Forward Network(FFN) for further feature extraction [24].

(1) Multi-Head Attention

MHA allows the model to learn attention weights in different representation subspaces in parallel. For a given input matrix, the calculation process of MHA is as follows.

Input $X_{T}$ is linearly transformed into Query (Q) matrix, Key (K) matrix, and Value (V) matrix.


$$\begin{array}{c} \text{Q}=X_{T}W^{Q},K=X_{T}W^{K},V=X_{T}W^{V} \end{array}$$ (8)

where Q, K, V represent Query matrix, Key matrix, and Value matrix, respectively. $\;X_{T}$

is the input matrix, $\;W^{Q}$, $W^{K}$ and $W^{V}$ are the corresponding weight matrices.

Q, K and V are split into h heads, each with dimension $d_{k}$, and attention is calculated in parallel for each head.


$$\begin{array}{c} Attention\left(Q_{i},K_{i},V_{i}\right)=softmax\left(\frac{Q_{i}K_{i}^{T}}{\sqrt{d_{k}}}\right)\text{V} \end{array}$$ (9)

where $d_{k}$ denotes the dimension of the vector, and softmax is used to calculate weights.

Finally, the outputs of all heads are concatenated and linearly transformed to obtain the final MHA output.


$$\begin{array}{c} MultiHead(Q,K,V)=Concat\left({head}_{\text{1}},{head}_{\text{2}},\ldots ,{head}_{h}\right)W^{O} \end{array}$$ (10)

Where ${head}_{i}$ represents the i−th attention head, $W^{O}$ is the weight matrix for output.

(2) Residual Connection and Layer Normalization

“Add & Norm” mechanism is applied after each sublayer in the Transformer encoder. Add represents Residual Connection to prevent degradation in deep networks. Norm represents Layer Normalization to normalize activation values for each layer. The equation is shown in (11).

The output of MHA undergoes residual connection and layer normalization.


$$\begin{array}{c} X_{T}^{\prime }=LayerNorm\left(X_{T}+MHA\left(X_{T}\right)\right) \end{array}$$ (11)

Where $X_{T}$ denotes the input of MHA, $MHA(X_{T})$ represents the output of MHA.

(3) Feed Forward Network

After Add & Norm of MHA output, it is fed into FNN. The FNN contains two linear transformations with ReLU activation function in between.


$$\begin{array}{c} X_{FFN}=ReLU\left(X_{T}^{\prime }\cdot W_{\text{1}}+b_{\text{1}}\right)\cdot W_{\text{2}}+b_{\text{2}} \end{array}$$ (12)

$$\begin{array}{c} X_{output}=LayerNorm\left(X_{T}^{\prime }+X_{FFN}\right) \end{array}$$ (13)

Where $X_{FFN}$ denotes the output of FNN, $X_{T}^{\prime }$ is the output after MHA, $X_{output}$ represents the output after Add & Norm. $\;W_{\text{1}}$ and $W_{\text{2}}$ are learnable weight matrices, $\;b_{\text{1}}$ and $b_{\text{2}}$ are bias vectors.

By stacking multiple such encoder layers, the Transformer can effectively capture deep global dependencies in the input sequence. MHA enables the model to attend to different positions of input sequence through parallel attention computation, thus learning complex long-distance dependencies. Meanwhile, positional encoding ensures that sequential order information is preserved when processing sequence data. These modules work together to enable the Transformer to perform deeper and more effective feature extraction in sequence prediction tasks, thereby enhancing its modeling capability and prediction accuracy.

## Transfer learning

TL aims to leverage source domain knowledge to improve prediction performance in target domain [25]. For regression problems, the source domain dataset is denoted as $D_{s}=\{X_{s},Y_{s}\}$ with sufficient labeled samples; the target domain dataset is denoted as $D_{t}=\{X_{t},Y_{t}\}$ with typically fewer samples. The regression function f(x) maps input features to continuous target values. The goal of TL is to improve the generalization ability of regression model f(x) in the target domain by learning the similarity between the source domain $D_{s}$ and the target domain $D_{t}$.

In predicting the corrosion rate of oil and gas pipelines, there are differences between pipelines under different operating conditions, but their corrosion mechanisms are similar. Key features such as oil pressure, CO_2_ partial pressure, H_2_S partial pressure, and pH value exhibit similar numerical distribution characteristics in $D_{s}$ and $D_{t}$. However, due to the distribution differences between domains, directly applying f(x) trained on $D_{s}$ to $D_{t}$ often results in larger prediction errors. Therefore, we adopt a model-based transfer strategy: first pre-train the model on $D_{s}$ to obtain initial parameters, then optimize the model on $D_{t}$ through gradual fine-tuning to improve the prediction accuracy of f(x) in the target domain. The entire transfer process is divided into two stages: source domain pre-training and target domain gradual fine-tuning.

### Pre-training

**Fig 3:**
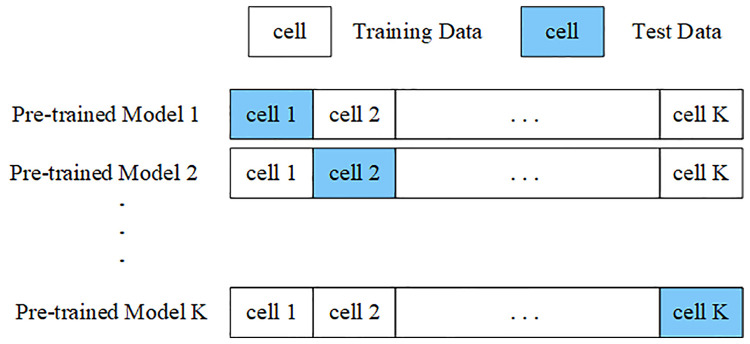
K-fold cross-validation.

During the pre-training process, K-fold cross-validation was employed to train the source domain data [26]. The source domain data is randomly divided into K mutually exclusive subsets, referred to as folds, followed by K conducting multiple rounds of model training. In each round, one fold was retained as the validation set, while the remaining K−1 folds were used for training. Each round of training retained a different fold as the validation set. The validation errors from each round were recorded, including the Mean Absolute Error(MAE), Root Mean Square Error (RMSE), and the Coefficient of Determination($R^{\text{2}}$). After completing K rounds of model training, the average error across all folds was computed, and the network structure and parameters of this model were subsequently used for TL. The K-fold cross-validation process is illustrated in Fig 3.

The model parameters from this stage are used as initial weights for fine-tuning in the target domain, allowing the model to avoid learning from scratch, thus accelerating convergence in the target domain.

### Gradual fine-tuning in the target domain

**Fig 4:**
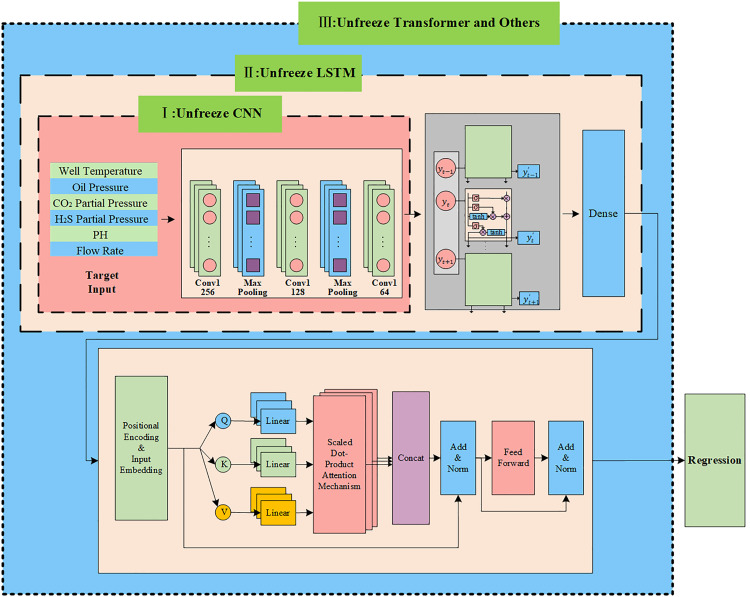
Gradual fine-tuning strategy.

Due to the distribution differences between the source domain and the target domain data, we introduce a Gradual fine-tuning strategy in the second phase of TL. First, all layers in the feature extraction model are frozen. Then, target domain data is fed into the model, and different parts of the model are gradually unfrozen for fine-tuning. The fine-tuning process in this study is divided into three stages: fine-tuning the CNN part, fine-tuning the LSTM part, and fine-tuning the Transformer and Dense layers, as illustrated in Fig 4.

**Stage 1:** Unfreeze the CNN layers for fine-tuning and update parameters only in these layers. The training at this stage adjusts the convolutional layers based on the target domain data, making the underlying feature extraction patterns more suitable for the target domain.
**Stage 2:** Unfreeze the LSTM layers for fine-tuning. The weights of the LSTM are gradually adjusted through gradient updates based on the target domain data, allowing it to capture the long-term dependencies within the target domain.
**Stage 3:** Unfreeze the Transformer module and the final Dense layer. The Transformer module further recalibrates features through the MHA. In this stage, the Transformer module is unfrozen to capture the global dependencies of the target domain data. The final Dense layer then further maps the features to the output space, making the model’s predictions more consistent with the actual data distribution in the target domain.

The detailed implementation process of the gradual TL strategy is shown in Table 1

**Table 1:** Pseudocode for the Gradual TL strategy.

**Algorithm 1:** CNN-LSTM-Transformer TL for Corrosion Rate Prediction. **Input:** Source data $\left\{X_{data\text{1}},Y_{data\text{1}}\right\}$, Target data $\left\{X_{data\text{2}},Y_{data\text{2}}\right\}$. **Parameter**: Number of epochs E=50, Batch size $n^{batch}$, Learning rateα. **Output**: Fine-tuned model with optimal parameters for target domain.
1: for epoch = 1 to m do # Loop over epochs for source domain training 2: Sample a batch of $\left\{X_{data\text{1}},Y_{data\text{1}}\right\}$ 3: # Update parameters using Adam optimizer Compute gradients $g_{\theta }=\nabla_{\theta }L(X_{data\text{1}},Y_{data\text{1}};\theta )$ 4: $m_{t}=\beta_{\text{1}}m_{t-\text{1}}+(\text{1}-\beta_{\text{1}})g_{t}\;v_{t}=\beta_{\text{2}}v_{t-\text{1}}+(\text{1}-\beta_{\text{2}})g_{t}^{\text{2}}$ 5: $\theta_{t}=\theta_{t-\text{1}}-\alpha \frac{m_{t}}{\sqrt{v_{t}+\in }}$ 6: end for 7: Freeze all layers in the feature extractor 8: i←1 9: while i≤3 do # Fine-tuning in stages on target domain if i=1: unfreeze CNN layers train on $\left\{X_{data\text{2}},Y_{data\text{2}}\right\}$ with Adam optimizer else if i=2: unfreeze LSTM layers train on $\left\{X_{data\text{2}},Y_{data\text{2}}\right\}$ with Adam optimizer else if i=3: unfreeze Transformer layers train on $\left\{X_{data\text{2}},Y_{data\text{2}}\right\}$ with Adam optimizer end if i←i+1 end while

## Experimental results and comparative analysis

### Dataset description

The data utilized in this research was obtained from a petroleum corporation in China. The operational environments of different oil and gas pipelines corrosion data vary significantly. However, their working principles are similar, which leads to correlations among corrosion characteristics. Dataset A, designated as the source domain, comprises 1,736 entries. Concurrently, Dataset B, serving as the target domain, contains 67 records. The current corrosion related parameters for oil and gas pipelines s encompass the following variables: Temperature, Oil pressure, CO₂ partial pressure, H₂S partial pressure, pH value, Flow velocity. Among these, temperature is the most critical factor influencing pipeline corrosion. The feature descriptions of the collected data are presented in Table 2, while the target domain data is summarized in Table 3.

**Table 2:** Dataset feature description.

No.	Parameter	Description
1	Temperature (TM)	Temperature of the pipeline
2	Oil Pressure (OP)	Pressure of oil in the pipeline
3	CO₂ Partial Pressure (PCO₂)	CO₂ partial pressure in corrosive medium
4	H₂S Partial Pressure (PH₂S)	H₂S partial pressure in corrosive medium
5	pH (pH)	Acidity level of the medium in pipeline
6	Flow Velocity (UG)	Flow velocity of fluid in the pipeline

**Table 3:** Target domain data.

	X1	X2	X3	X4	X5	X6
No.	TM(°C)	OP(MPa)	PCO_2_(Pa)	PH_2_S(Pa)	pH	UG(m/s)
1	87	150.7	2.5139	0.1172	6.39	1.89
2	70	142	3.6681	8.483	6.8	2.15
3	87	150.7	2.5139	0.1172	6.39	1.89
…	…	…	…	…	…	…
67	73.5	32	0.3874	0.0877	6	2.51

Table 4 presents the descriptive statistics of the dataset features, including mean, variance(var), standard deviation(std), minimum(min), and maximum(max) values for each variable (X1 to X6). These statistics summarize the central tendency, dispersion, and range of each feature, providing insight into their distribution characteristics. Prior to model training, the data underwent preprocessing steps including standardization and normalization to ensure consistent feature scaling and improve model performance.

**Table 4:** Statistical summary of key features.

	mean	var	std	min	max
X1	84.74	433.69	20.82	36.27	105.0
X2	94.91	2239.78	47.32	32.0	151.10
X3	1.65	1.24	1.11	0.38	3.66
X4	1.25	7.89	2.80	0.001	8.48
X5	6.11	0.20	0.45	5.40	7.0
X6	2.40	0.60	0.77	1.82	5.03

### Experimental procedures

#### Experimental design.

In this research, we designed two experiments to evaluate the performance of the CNN-LSTM-Transformer model in predicting oil and gas pipelines corrosion rates. **Case 1:** Compare the performance of the CNN-LSTM-Transformer model under different TL strategies, including Non-transfer, Direct transfer, and Gradual transfer. Non-transfer involves training and predicting the model directly on the target domain data without any TL. Direct transfer refers to fine-tuning, training, and predicting the model on the target domain data using traditional TL. Gradual transfer comprises training and predicting the model using the transfer strategy proposed in this study. **Case 2:** We compared the CNN-LSTM-Transformer model with other TL models to validate the effectiveness of our proposed algorithm. This series of experiments aims to comprehensively evaluate the model’s performance and its advantages in practical applications.

#### Experimental environment.

Different experimental platforms may introduce unnecessary variability into the results. Therefore, the same computer is used to ensure the fairness and validity of the results. The computer configuration used in this experiment is an AMD Ryzen 9 5900HS processor with Radeon Graphics @ 3.30 GHz, 16 GB of RAM, Nvidia GeForce RTX 3060 GPU, TensorFlow 2.6.0 platform, Windows 11 operating system, and Python 3.9.12.

#### Experimental parameter settings.

In the training and prediction process of the model, numerous hyper-parameters are involved, and their settings significantly influence the final prediction results. After multiple rounds of pre-training and optimization adjustments, the parameter settings are presented in Table 5.

**Table 5:** Architectural configuration parameters of CNN-LSTM-Transformer.

Network Layer	Filter Size	Filters	Stride	Weights	Biases
Conv 1	2	256	1	2 × 256 + 256	256
MaxPool 1	2	–	2	–	–
Conv 2	2	128	1	256 × 2 × 128 + 128	128
MaxPool 2	2	–	2	–	–
Conv 3	2	64	1	128 × 2 × 64 + 64	64
LSTM	–	50	–	4×(64 + 50)×50	200
FC1	–	50	–	50 × 50 + 50	50
Transformer	–	64	–	4×(50 × 50)+50	200
FC2	–	256	–	50 × 256 + 256	256

#### Evaluation metrics.

This study employs three metrics MAE, RMSE,$R^{\text{2}}$, Mean Absolute Percentage Error(MAPE), and Root Mean Square Percentage Error (RMSPE) to evaluate the experimental results. The mathematical formulations of these evaluation metrics are presented in Equations (14)-(18).


$$\text{MAE}=\frac{\text{1}}{n}\sum_{i=\text{1}}^{n}\left|{\hat{y}}_{i}-y_{i}\right|$$ (14)

$$\text{RMSE}=\sqrt{\frac{\text{1}}{n}\sum_{i=\text{1}}^{n}{\left({\hat{y}}_{i}-y_{i}\right)}^{\text{2}}}$$ (15)

$$\begin{array}{c} R^{\text{2}}=\text{1}-\frac{\sum_{i}({{\hat{y}}_{i}-y_{i})}^{\text{2}}}{\sum_{i}({{\hat{y}}_{i}-\overset{―}{y})}^{\text{2}}} \end{array}$$ (16)

$$\text{MAPE}=\frac{\text{1}\text{0}\text{0}\%}{n}\sum_{i=\text{1}}^{n}\left|\frac{{\hat{y}}_{i}-y_{i}}{y_{i}}\right|$$ (17)

$$\text{RMSPE}=\sqrt{\frac{\text{1}}{n}\sum_{i=\text{1}}^{n}{\left(\frac{{\hat{y}}_{i}-y_{i}}{y_{i}}\right)}^{\text{2}}}\times \text{1}\text{0}\text{0}\%$$ (18)

where  n represents the number of predicted samples, $y_{i}$ denotes the true value, $\hat{y_{t}}$ represents the model’s predicted value, and $\overset{―}{y}$ denotes the mean of true values.

### Results and analysis

#### Comparative analysis of different transfer strategies.

The dataset from target domain B was partitioned into training and testing sets with a 7:3 ratio. As shown in Table 6, the three distinct transfer strategies demonstrated significant performance variations in predicting pipeline corrosion rates. Specifically, CNN-LSTM-Transformer-N, which directly performs predictions on the target domain without any TL, exhibited the poorest performance with an MAE of 0.051, RMSE of 0.063, and $R^{\text{2}}$ of 0.610. These metrics indicate substantial errors in capturing pipeline corrosion characteristics, suggesting insufficient adaptation to the target domain’s data distribution. In contrast, CNN-LSTM-Transformer-S, implementing direct transfer with simple fine-tuning on the target domain training set, showed marked improvement over the non-transfer approach. This strategy achieved reduced MAE and RMSE values of 0.030 and 0.039 respectively, with an enhanced $R^{\text{2}}$ of 0.850. These improvements demonstrate that even basic TL and fine-tuning can significantly enhance model performance and domain adaptation capabilities.

**Table 6:** Comparative analysis of different transfer strategies.

Algorithm	MAE	RMSE	$R^{2}$	MAPE	RMSPE	Times(s)
CNN-LSTM-Transformer-N	0.051	0.063	0.610	48.64%	71.72%	3.26
CNN-LSTM-Transformer-S	0.030	0.039	0.850	53.35%	77.27%	45.51
CNN-LSTM-Transformer-Y	**0.021**	**0.031**	**0.909**	**54.21%**	**81.12%**	**47.79**

**Fig 5:**
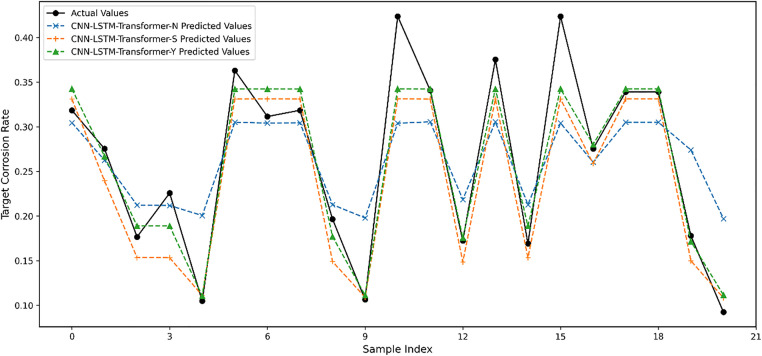
Impact of transfer strategies on predictions.

Most notably, CNN-LSTM-Transformer-Y, employing a Gradual transfer strategy from source to target domain with Gradual fine-tuning, achieved superior performance. This approach yielded the lowest MAE of 0.021 and RMSE of 0.031, while achieving an $R^{\text{2}}$ of 0.909. These metrics indicate that the Gradual transfer and Gradual fine-tuning methodology effectively leverages source domain knowledge while optimizing adaptation to target domain characteristics, resulting in substantially improved prediction accuracy. The prediction curves in Fig 5 corroborate these findings, showing that CNN-LSTM-Transformer-Y’s predictions closely align with actual values, demonstrating its significant advantages in capturing both the temporal trends and volatility patterns of pipeline corrosion rates.

#### Comparison with other TL models.

**Fig 6:**
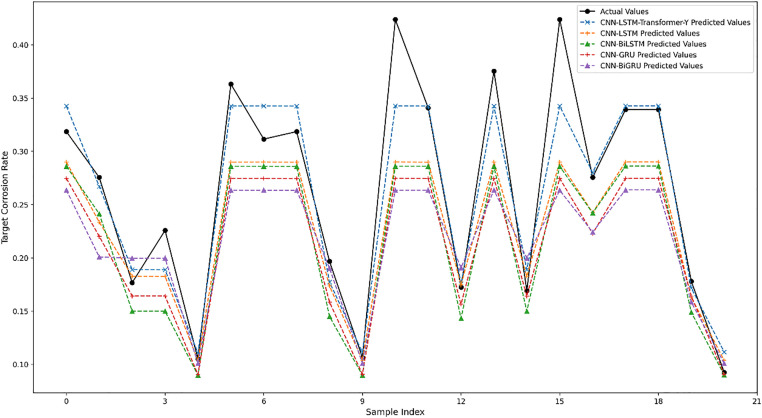
Impact of alternative TL models.

To validate the effectiveness of our proposed TL model, we conducted comparative experiments with alternative TL strategies. As demonstrated in Table 7 and Fig 6, distinct TL approaches exhibited notable performance variations in pipeline corrosion rate prediction. The first four models, employing simple fine-tuning strategies, showed relatively higher prediction errors. Specifically, CNN-LSTM [22] achieved an MAE of 0.040, RMSE of 0.055, and $R^{\text{2}}$ of 0.705, demonstrating relatively superior performance among the four simple fine-tuning approaches. In contrast, CNN-BiLSTM [27], CNN-GRU [28], and CNN-BiGRU [29] exhibited higher MAE and RMSE values with progressively decreasing $R^{\text{2}}$ values, particularly BiGRU with an $R^{\text{2}}$ of merely 0.489, indicating limited adaptation capability to target domain data.

**Table 7:** Comparison of alternative TL models.

Algorithm	MAE	RMSE	$R^{2}$	MAPE	RMSPE	Times(s)
CNN-LSTM	0.040	0.055	0.705	47.93%	66.29%	43.17
CNN-BiLSTM	0.048	0.061	0.643	49.56%	66.24%	57.10
CNN-GRU	0.052	0.066	0.572	47.52%	62.84%	41.40
CNN-BiGRU	0.056	0.073	0.489	45.94%	60.22%	58.00
CNN-LSTM-Transformer-Y	**0.021**	**0.031**	**0.909**	**54.21%**	**81.12%**	**47.79**

In contrast, the CNN-LSTM-Transformer-Y model, implementing Gradual fine-tuning, significantly outperformed other models with an MAE of 0.021, RMSE of 0.031, and $R^{\text{2}}$ reaching 0.909. The Gradual fine-tuning strategy enabled the model to effectively extract valuable knowledge from the source domain while gradually adapting to target domain characteristics. This superiority is further evidenced in Fig 6, where the CNN-LSTM-Transformer-Y prediction curve shows high concordance with actual values, substantially surpassing models using simple fine-tuning. Therefore, the Gradual fine-tuning strategy demonstrates enhanced generalization capability and prediction accuracy in TL, particularly exhibiting significant advantages in scenarios with limited target domain data.

Fig 6 illustrates the prediction performance of different transfer learning models by comparing predicted corrosion rates against actual values. Notably, the CNN-LSTM-Transformer-Y model achieves the closest alignment with the ground truth curve, visually confirming its superior predictive accuracy. In contrast, other models show larger deviations, particularly in regions with rapid changes, highlighting the effectiveness of the Gradual fine-tuning strategy in improving temporal prediction fidelity.

## Validation

To further validate the generalization capability of our proposed model, we conducted additional comparative experiments using publicly available datasets from Ryerson University and Memorial University of Newfoundland. Table 8 datasets [5] contain 243 samples of CO_2_ corrosion rate simulation data, including key features such as temperature of fluid, internal pressure, CO_2_ partial pressure, flow velocity inside the pipe, and corrosion inhibitor efficiency. Our experimental results demonstrate that the proposed model achieves MAE of 0.328,RMSE of 0.041,${\text{R}}^{\text{2}}$ of 0.866, MAPE of 31.16% and RMSPE of 41.51%, indicating strong predictive performance and excellent generalization ability.

**Table 8:** Performance comparison on public CO_2_ corrosion dataset.

Algorithm	MAE	RMSE	$R^{2}$	MAPE	RMSPE	Times(s)
CNN-LSTM-Transformer-N	0.479	0.624	0.702	29.16%	37.07%	3.94
CNN-LSTM-Transformer-S	0.897	1.107	0.001	22.57%	28.91%	12.96
CNN-LSTM-Transformer-Y	**0.328**	**0.419**	**0.866**	**31.16%**	**41.51%**	**15.12**

## Conclusion

Through comparative analysis of different experimental results, this study has yielded several definitive conclusions. First, experiments with three different transfer strategies demonstrated that the Gradual transfer strategy (CNN-LSTM-Transformer) outperformed both Non-transfer and Direct transfer approaches in terms of prediction accuracy and generalization capability. This model achieved an MAE of 0.021, RMSE of 0.031, and $R^{\text{2}}$ of 0.909, significantly surpassing other strategies and highlighting the effectiveness of Gradual fine-tuning in adapting to target domain data.

Furthermore, when comparing CNN-LSTM-Transformer with other TL models, we observed that the CNN-LSTM-Transformer model maintained substantial advantages. The Gradual fine-tuning enabled effective knowledge extraction from the source domain while gradually adapting to target domain characteristics, resulting in enhanced prediction accuracy and generalization capability. This approach was particularly effective in scenarios with limited target domain data. Overall, through systematic comparison of different models and transfer strategies, this study validates the superiority of CNN-LSTM-Transformer in pipeline corrosion rate prediction, indicating its promising potential for practical applications in pipeline corrosion management and maintenance.

Despite these promising results, some limitations should be acknowledged. First, the target dataset used in this study is relatively small (67 samples) and sourced from a single industrial partner, which may limit the generalizability of the findings. Nonetheless, the proposed approach was specifically designed for small-sample learning and demonstrated strong predictive performance even under data-scarce conditions. Second, the CNN-LSTM-Transformer model introduces added computational complexity compared to simpler alternatives; however, the significant performance gains achieved justify this trade-off in many real-world applications. In future work, we plan to validate the proposed method using larger and more diverse datasets from different operational environments, and to explore model optimization techniques that may reduce training overhead without compromising accuracy.
